# Optimization of Fermentation Conditions for Carrageenase Production by *Cellulophaga* Species: A Comparative Study

**DOI:** 10.3390/biology10100971

**Published:** 2021-09-27

**Authors:** Md Musa Howlader, Jana Molz, Nico Sachse, Rando Tuvikene

**Affiliations:** 1 School of Natural Sciences and Health, Tallinn University, Narva mnt 29, 10120 Tallinn, Estonia; mdmusa@tlu.ee (M.M.H.); jana.molz@uni-ulm.de (J.M.); nico.sachse@uni-ulm.de (N.S.); 2 Department of Biosciences, Institute of Pharmaceutical Biotechnology, Ulm University, Helmholtzstraße 16, 89081 Ulm, Germany

**Keywords:** *Cellulophaga*, ι-carrageenase, optimization, carrageenan, fermentation, enzyme

## Abstract

**Simple Summary:**

*Cellulophaga* species are rarely studied marine bacteria with the potential for carrageenase production. We examined the carrageenase secretion ability of six bacterial species from the *Cellulophaga* genus. Among them, *C. algicola* produced the maximum amount of ι-carrageenase. Most of the bacteria produced their highest quantity of enzymes at 25 °C after 48 h of incubation time. The maximum enzyme production was achieved with the fermentation medium composition of 30 g/L sea salt, 1.4 g/L furcellaran and 3 g/L yeast extract. In addition, the properties of the ultrafiltered ι-carrageenase extracted from *C. algicola* were studied.

**Abstract:**

Carrageenases appear in various species of marine bacteria and are widely used for the degradation of carrageenans, the commercially significant sulphated polysaccharides. The carrageenase production ability of six different *Cellulophaga* species was identified, with ι-carrageenase being the most abundant carrageenolytic enzyme. *C. algicola* was the most potent strain, followed by *C. fucicola* and *C. geojensis*, whereas *C. pacifica* was the least effective carrageenase producer among the studied strains. The enzyme production was maximized using the one-factor-at-a-time optimization method. The optimal incubation temperature was identified as 25 °C and the incubation time was set as 48 h for all tested species. The optimal medium composition for *Cellulophaga* strains was determined as 30 g/L sea salt, 1.4 g/L furcellaran, and 3 g/L yeast extract. An ultrafiltered enzyme extracted from *C. algicola* had the highest activity at around 40 °C. The optimal pH for enzymatic degradation was determined as 7.8, and the enzyme was fairly stable at temperatures up to 40 °C.

## 1. Introduction

Carrageenans are sulphated linear polysaccharides with a high molecular weight extracted from the cell wall components of the red algae (Rhodophyceae) [1]. Renowned for their unique gelling properties, the chemical structure of carrageenans is based on an alternating disaccharide repeating units of 1,3-linked β-ᴅ-galactose and 1,4-linked 3,6-anhydro-α-ᴅ-galactose [2]. Based on the presence of a 3,6-anhydro bridge in the 4-linked α-galactose residues and the number and position of sulphated substitutions, there are three major commercially important types of carrageenans: κ-, ι-, and λ-carrageenans [3]. Due to their unique physicochemical properties, carrageenans are widely used in the food, pharmaceutical, textile, and biotechnological industries [4,5]. Besides, they have been reported as having promising bioactivities, such as antiviral [6], antioxidant [7], anticoagulant [8], and gastroprotective [9] effects. The carrageenan oligosaccharides are also renowned for their potential for antiviral and antitumor [10], immunomodulatory [11], anti-inflammatory, and antioxidant [12,13] activities.

The preparation of carrageenan oligosaccharides is usually conducted by chemical hydrolysis [14], enzymatic modification [15], or radiation [16]. Chemical hydrolysis (e.g., acid hydrolysis using H₂SO₄ or HCl) [17] is the most used technique to obtain carrageenan oligosaccharides, owing to its considerable advantages. However, such chemical treatments are not environmentally friendly methods. Besides, chemical hydrolysis also possesses some risks, such as the removal of the sulphate ester groups and the production of unwanted by-products, which limits its industrial applications [18]. On the other hand, the enzymatic modification is a highly substrate-specific, efficient, and controllable method, which makes it more desirable than chemical hydrolysis [18,19]. Enzymes with the ability to hydrolyze major commercial types of carrageenans are called κ-, ι-, and λ-carrageenases. These enzymes belong to the GH (glycoside hydrolase) families and commonly cleave the internal β-1,4 glycosidic linkages of carrageenans [19]. Various marine bacteria, such as *Pseudomonas* sp. [20], *Vibrio* sp. [21], *Cytophaga* sp. [22], and *Cellulophaga* sp. [23], have been reported as carrageenase sources.

The genus *Cellulophaga* accommodates yellow/orange pigmented, gram-negative, strictly aerobic, rod-shaped, heterotrophic, and gilding bacterial species originating from distinct marine environments [24]. Proposed by Johansen et al., the genus belongs to the family Flavobacteriaceae of the phylum Bacteroidetes and consists of seven major species [25]: *C. lytica*, *C. fucicola*, *C. baltica* [26], *C. geojensis* [27], *C. algicola* [28], *C. tyrosinoxydans* [29], and *C. pacifica* [24]. Among them, *C. baltica* has previously been reported for having ι-carrageenase secretion abilities [15]. In addition, a novel cloned κ-carrageenase from *C. lytica* strain N5-2 could also degrade κ-carrageenan into neo-κ-carrahexaose and neo-κ-carraoctaose [23]. These reports emphasize the potential of *Cellulophaga* species becoming a reliable source of marine polysaccharide-degrading enzymes. Since research on the fermentation conditions of *Cellulophaga* species is scarce, knowledge of the optimal fermentation conditions of these widespread and rarely studied species of marine bacteria will contribute to the production of carrageenases on a large scale and could ensure their efficient use in industrial applications.

In the present study, the carrageenase activity of all six remaining species in the *Cellulophaga* genus was investigated, except *C. baltica*, which was revealed in our previous study. In addition, a comparison of optimal fermentation conditions for carrageenase production by these strains is presented and enzymatic degradation properties of an ultrafiltered ι-carrageenanse were determined.

## 2. Materials and Methods

### 2.1. Materials

κ-carrageenan (C1804), ι-carrageenan (C1805), and λ-carrageenan (C3313) were purchased from Tokyo Chemical Industry Co., Ltd., Tokyo, Japan. Furcellaran (Estgel 1000) was kindly provided by Est-Agar AS, Saaremaa, Estonia. Growth media components of different origins were used: agar (MC002) was purchased from Neogen Food Safety, sea salt (Instant Ocean; 97-2433) from Aquarium Systems, tryptone (BP9726-2) from Fisher Scientific, and yeast extract (Y0875) from Sigma-Aldrich.

The chemicals used for the determination of enzyme activity were as follows: Tris(hydroxymethyl)aminomethane (252859), Potassium hexacyanoferrate(III) (244023), Na_2_CO_3_ (4154566), and NaOH (415413) from Sigma-Aldrich. Bradford reagent (J61522) was purchased from Alfa Aesar. All the other necessary chemicals and reagents were supplied by Sigma-Aldrich.

### 2.2. Methods

#### 2.2.1. Bacterial Strains

The *Cellulophaga* species originated from various marine habitats of the world and were supplied by DSMZ (Germany). *C. algicola* was collected from the surface of sea ice diatoms at Antarctica in 2000 (GenBank accession no. NR028661); *C. fucicola* from the surface of brown alga *Fucus serratus* L., at Kattegat, Denmark in 1999 (GenBank accession no. NR025287); *C. lytica* from the sandy beach mud at Harwich, Eastern Coast, in 1981 (GenBank accession no. NR074464); *C.* *tyrosinoxydans* from seawater of the eastern coast at Jeju Island, Republic of Korea in 2007 (GenBank accession no. NR044502), *C. geojensis* from marine sand at Geoje, South Korea in 2011 (GenBank accession no. NR118002); and *C. pacifica* from sea water of Amursky Bay at Gulf of Peter the Great, Sea of Japan, Pacific Ocean in 2000 (GenBank accession no. NR114003). Upon arrival at our laboratory, the samples were cultured on agar plates consisting of 15 g/L agar, 30 g/L sea salt, 5 g/L tryptone, 0.8 g/L furcellaran, and 1 g/L yeast extract, and kept at room temperature.

#### 2.2.2. Optimization of Culture Medium by One-Factor-at-a-Time Experiment

The seed culture medium was prepared in 14 mL BD falcon round-bottom tubes consisting of 30 g/L sea salt (Cl^−^ 47.47%, Na^+^ 26.28%, SO_4_^2^^−^ 6.6%, Mg^2+^ 3.23%, Ca^2+^ 1.01%, K^+^ 1.02%, HCO_3_^−^ 0.491%) [30], 1.6 g/L furcellaran, 4 g/L yeast extract, and pH 7.5. Each tube contained 4 mL of liquid medium. The same medium composition was used in 250 mL Erlenmeyer baffled flask containing 50 mL of liquid as a fermentation medium for carrageenase production. The first experiment of this study (optimal substrate specificity) was performed with this medium. Later, the composition of the fermentation medium was altered by the one-factor-at-a-time method to determine the optimal fermentation condition where six independent variables were examined: growth temperature and incubation time, sea salt concentration, nitrogen source, yeast extract, and furcellaran concentrations [31]. These variables were fixed at a specific value. Only one variable was changed at a time, and variables were changed chronologically to determine the optimal value. The optimal value was then used for the following experiments [15,31].

#### 2.2.3. Bacterial Growth Conditions

The seed culture medium tubes were inoculated with different *Cellulophaga* species and incubated overnight in a WIS-30 Shaking incubator (Witeg, Wertheim, Germany) at 20 °C and 160 rpm. Bacterial growth was monitored by a DSM-Micro cell density meter (Lab net, Mayfield Ave Edison, NJ, USA). When the cell density reached at 0.4 (Abs), 300 µL of the seed culture was inoculated into sterile fermentation medium flasks for incubation. The incubation time and temperature were 72 h and 20 °C for the substrate specificity experiment, respectively, and were later optimized for the next experiments. The flask caps were placed loosely during the incubation period to ensure sufficient oxygen supply and covered by aluminium foil to prevent contamination. Cell density was measured after 48 h of incubation, and the culture broth was centrifuged at 4 °C and 10,000× *g* for 10 min [15]. After centrifugation, the collected supernatants (crude enzymes) were kept on ice and used for the following experiment.

#### 2.2.4. Determination of Carrageenase Activity

The carrageenase activity of the obtained supernatants was determined by mixing 85 μL of the crude enzyme with 85 μL of 0.1% substrate solution in 20 mM Tris–HCl (pH 7.8) in 2 mL screw-cap Eppendorf tubes. The mixtures were then incubated at 40 °C and 1000 rpm using a TS-100 thermo-shaker (Biosan, Riga, Latvia) for 30 min and heated at 99.5 °C for 20 min without shaking to inactivate the enzyme. Ferricyanide assay was used to determine the amount of reducing sugars released by the substrate solution inside the reaction tubes [32]. Under the abovementioned reaction conditions, the amount of enzyme required to release one μmole of ᴅ-galactose per min was described as one unit of carrageenase activity [33]. In addition, for the blank, the crude enzyme was inactivated by heating at 99.5 °C for 20 min before the reaction. Total protein concentration was determined by the Bradford assay where bovine serum albumin was used as a standard [34]. For the first experiment (optimal substrate specificity), nine different types of polysaccharides (κ-, ι-, λ-, and θ carrageenans; furcellaran; agarose; galactan mixture from *Gigartina radula*; funoran; and sulphated chitosan) were used to prepare the substrate solution. ι-carrageenan was selected as the most suitable substrate, which was then used for all the remaining experiments.

#### 2.2.5. Effect of Temperature and pH on Enzymatic Degradation

Upon the determination of the optimal fermentation conditions, a set of new media was prepared using the optimal conditions, and 50 mL crude enzymes of all six bacteria were extracted by the procedure mentioned in Section 2.2.3. After that, the collected crude enzymes were filtered, washed, and concentrated to 2 mL using the Sartorius Stedium ultrafiltration device (10,000 MWCO) and 20 mM Tris–HCl buffer solution (pH 7.8). The collected enzymes were then kept on ice for the next step.

The optimal temperature of enzymatic degradation was determined at incubation temperatures of 15, 20, 25, 30, 35, 40, 45 and 50 °C. For thermostability measurement, the enzymes were preheated at different temperatures, ranging from 10 °C to 70 °C, for 30 min, and cooled down by keeping on ice. After that, 50 μL enzyme solutions were added into 500 μL 0.1% ι-carrageenan solution in 20 mM Tris–HCl buffer (pH 7.8) in 2 mL screw-cap Eppendorf tubes and incubated with shaking for 180 min. In addition, for the determination of optimal pH, 0.1% ι-carrageenan substrate solutions were prepared in 20 mM Tris–HCl buffer with different pH values (6.5, 7.0, 7.5, 7.8, 8.0, 8.5, 9.0, and 9.5) and used for the enzymatic hydrolysis. The incubation was carried out in TS-100 thermo-shakers (Biosan, Riga, Latvia), and the enzymes were inactivated by heating the solutions at 99.5 °C for 20 min. The solutions were then cooled down at room temperature, mixed with 0.1 M NaNO_3_, and filtered by 0.22 μm CA filters for size-exclusion chromatography analysis.

#### 2.2.6. Size-Exclusion Chromatography

A Shimadzu LC-30AD liquid chromatograph equipped with a RID-10A refractive index detector and Shimadzu CTO-20AC column oven were used for the size-exclusion chromatography analysis. First, 100 μL of the filtered samples were passed through an OHpak SB-G guard column and two Shodex OHpak SB-806MHQ columns in series. The elution was conducted using 0.1 M NaNO_3_ solution as the mobile phase at a flow rate of 0.8 mL/min. The column oven temperature was maintained at 60 °C. A calibration curve obtained from 12 pullulan standards (Mp values of 2400, 1560, 710, 380, 200, 106, 45.9, 22.0, 11.2, 5.6, 1.08, and 0.342 kDa) was used for the estimation of the peak average, weight average, and number average molecular weights by the LabSolutions software.

#### 2.2.7. Statistical Analysis

GraphPad Prism software (Version 9.1.1; San Diego, CA, USA) was used for statistical analysis. A two-way ANOVA test was conducted to observe the significant difference between test parameters of each species. Multiple comparisons between the significant levels of interactions of the variables were conducted using Tukey’s test. Values were expressed as the mean ± SEM (n = 4), and *p* < 0.05 was considered as the presence of a statistically significant difference.

## 3. Results and Discussion

### 3.1. Phylogeny Analysis

**Figure 1:**
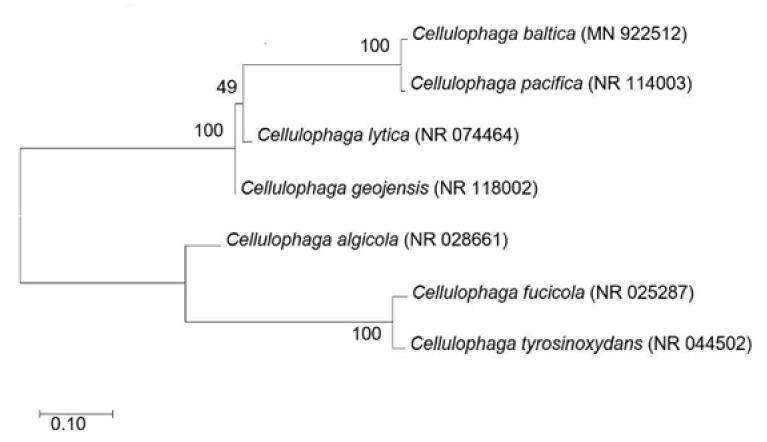
Phylogenetic analysis of the major *Cellulophaga* species. The phylogenetic tree was constructed using MEGA 4.0 software via the neighbor-joining method based on 16S rRNA gene sequences. The sequences were aligned using ClustalX. Numbers after the strain names refer to GenBank accession numbers of 16S rRNA sequences.

The phylogenetic tree (Figure 1) shows the similarity between the different *Cellulophaga* species used in this study, along with *C. baltica*, based on their 16S rRNA gene sequences obtained from GenBank. Although their habitat (as described in the Section 2.2.1) differs from each other to a great extent, *C. baltica* and *C. pacifica* were closely related with each other. The same scenario was also observed between *C. fucicola* and *C. tyrosinoxydans*. In further experiments, some bacteria showed similar carrageenase production patterns based on growth components and parameters, which may be attributed to their close relation in the phylogenetic tree.

### 3.2. Optimal Substrate Specificity

**Figure 2:**
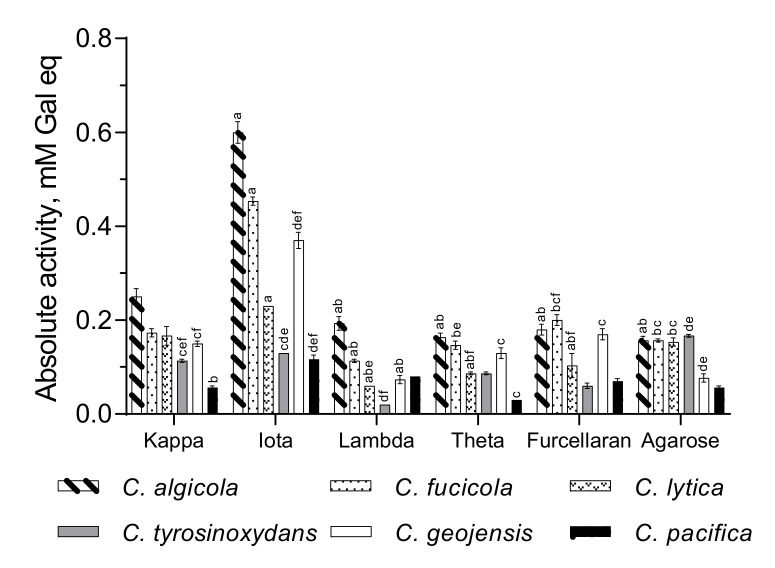
Substrate specificity of crude enzymes from the different *Cellulophaga* species and absolute activity as the galactose concentration (mM) equivalent. Values are expressed as mean ± SEM (n = 4); Data comparison was performed against different substrates. *a* = when compared with kappa carrageenan, *b* = when compared with iota carrageenan, *c* = when compared with lambda carrageenan, *d* = when compared with theta carrageenan, *e* = when compared with furcellaran, *f* = when compared with agarose. Different lowercase letters on the bars indicate statistically significant differences from respective group using ANOVA, followed by the Tukey’s comparison test (*p* < 0.05).

The cell-free culture supernatants were tested against nine polysaccharides, and notable enzyme activity was observed against the major carrageenans (κ-, ι-, λ-, and θ carrageenans, furcellaran) and agarose. The collected crude enzymes did not exhibit significant activity against polysaccharides, such as the galactan mixture from *Gigartina radula*, funoran, and sulphated chitosan (data not shown). As presented in Figure 2, five tested species showed higher ι-carrageenase secretion ability. *C. tyrosinoxydans* revealed higher agarase production but could also effectively hydrolyze ι-carrageenan. This phenomenon indicates that the collected supernatants of these *Cellulophaga* species contained ι-carrageenase. The fermentation media of *C. algicola* had the highest absolute activity (~0.4 mM galactose equivalent) among the tested species against ι-carrageenan, followed by those of *C. fucicola* and *C. geojensis*. Muzyed et al. also reported the extraction and purification of a ι-carrageenase from *C. baltica*, a marine bacterium from the *Cellulophaga* genus [15]. Ma et al. also revealed the degradation of both κ- and ι-carrageenan by *Cellulophaga* sp. QY3 [35]. Nevertheless, only ι-carrageenan was used as a substrate in the follow-up experiments, even though the possibility that more than one substrate could be degraded by one *Cellulophaga* species.

### 3.3. Optimal Growth Temperature and Incubation Time

**Figure 3:**
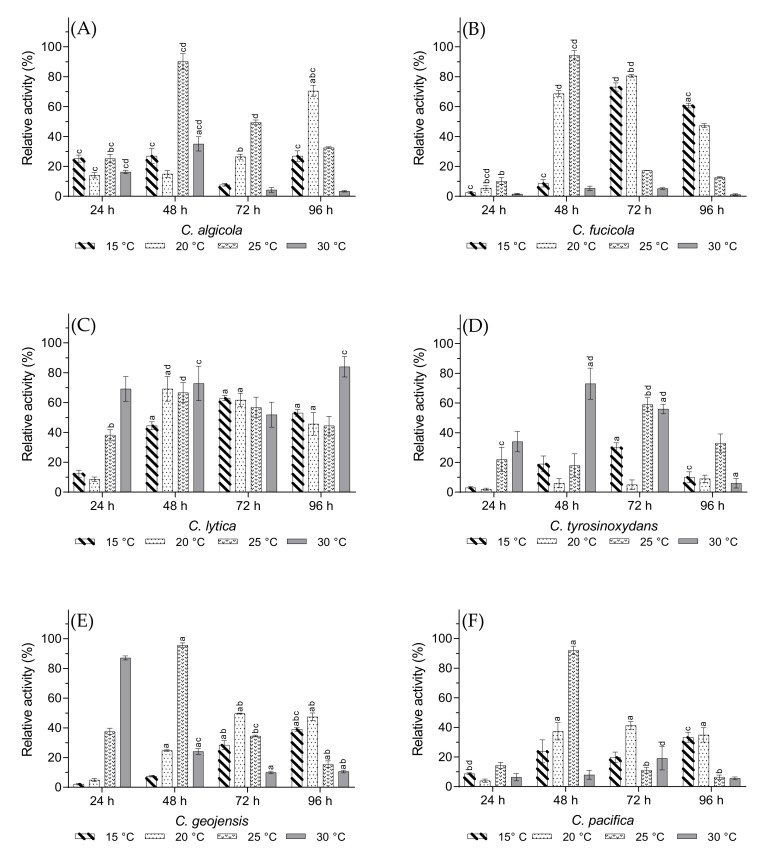
The effects of incubation time and temperature on carrageenase production from (**A**) *C. algicola*, (**B**) *C. fucicola*, (**C**) *C. lytica*, (**D**) *C. tyrosinoxydans*, (**E**) *C. geojensis*, and (**F**) *C. pacifica*. Values are expressed as mean ± SEM (n = 4). Data comparison was performed against different incubation time. *a* = when compared with 24 h, *b* = when compared with 48 h, *c* = when compared with 72 h, *d* = when compared with 96 h. Different lowercase letters on the bars indicate statistically significant differences from respective group using ANOVA, followed by Tukey’s comparison test (*p* < 0.05).

Carrageenase production by the *Cellulophaga* species greatly depends on the incubation time and temperature. Muzyed et al. determined the optimal incubation time and temperature as 48 h and 20 °C, respectively, for ι-carrageenase production by *C. baltica*, with a similar medium composition as in the present study [15]. In addition, Xiao et al. reported maximum carrageenase production at approximately 40 h and 20 °C for *Pseudoalteromonas carrageenovora* ASY5, a marine bacterium belonging to a the Proteobacteria phylum [31]. As shown in Figure 3, the majority of the tested *Cellulophaga* species produced the maximum amount of ι-carrageenase after 48 h at 25 °C, except *C. lytica* and *C. tyrosinoxydans*. Although the optimal incubation time was 48 h for *C. tyrosinoxydans*, it produced the highest amount of ι-carrageenase at 30 °C, similarly to *C. lytica*. However, *C. tyrosinoxydans* required 96 h of incubation time. Ma et al. stated that the optimal incubation time and temperature range for carrageenase production also led to the maximum production of other metabolic enzymes [35]. Therefore, carrageenase synthesis is a part of a complex metabolic network that has developed in a species-specific manner based on natural living conditions. Considering that the habitat of *C. lytica* is in the aquatic coastline of the Caribbean Sea with an average water temperature of 29 °C [36], these natural living conditions were probably the key factor behind the stable production rate of carrageenase at 30 °C with an optimal value even after 96 h. This factor may also be attributed to *C. tyrosinoxydans*, which belongs to the warm water habitats around South Korea where the water temperature varies between 14–27 °C [37]. *C. tyrosinoxydans* showed an optimal incubation temperature of 30 °C in this study.

### 3.4. Optimal Sea Salt Concentration

**Figure 4:**
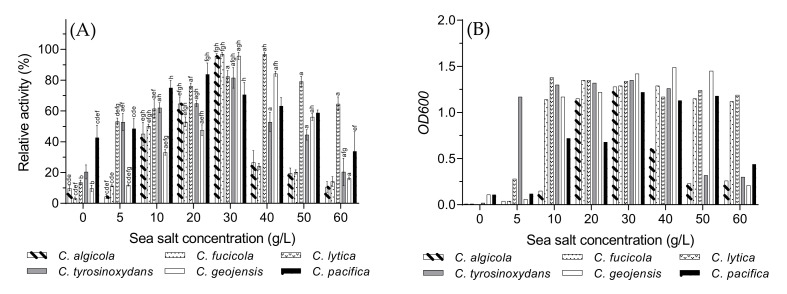
The effects of different sea salt concentrations on *Cellulophaga* species on (**A**) ι-carrageenase production and (**B**) cell growth presented as optical density (*OD_600_*). Values of Figure 4A are expressed as mean ± SEM (n = 4). Data comparison was performed against sea salt concentrations. *a* = when compared with 0 g/L, *b* = when compared with 5 g/L, *c* = when compared with 10 g/L, *d* = when compared with 20 g/L, *e* = when compared with 30 g/L, *f* = when compared with 40 g/L, *g* = when compared with 50 g/L, *h* = when compared with 60 g/L. Different lowercase letters on the bars indicate statistically significant differences from respective group using ANOVA, followed by Tukey’s comparison test (*p* < 0.05).

The *Cellulophaga* species needed an adequate amount of sea salt in the fermentation medium to produce ι-carrageenase. As shown in Figure 4A, the effect of sea salt concentration on ι-carrageenase production by *Cellulophaga* strains was examined at the range of 0–60 g/L, and the optimal range was 20–40 g/L, which was much higher than a previously reported bacteria *C. baltica* [15]. Originated from the Baltic Sea, *C. baltica* needed 5 g/L of sea salt in the fermentation medium to produce ι-carrageenase. The brackish water habitat of the Baltic Sea may be the possible reason for such a low sea salt tolerance [15]. However, the majority of species produced the highest amount of carrageenase at 30 g/L sea salt. The optimal value for *C. pacifica* was 20 g/L and for *C. lytica* 40 g/L. On the other hand, the enzyme production rates of *C. algicola* and *C. fucicola* followed similar patterns in different sea salt concentrations and decreased rapidly after their optimal concentrations (30 g/L). Although their growth patterns, as shown in Figure 4B, were quite different from each other, both species were close to each other in the phylogenetic tree (Figure 1). This could explain the reason behind their similar patterns of enzyme production and the sudden drop after reaching the maximum. However, the bacterial species did not produce much carrageenase without sea salt or at the lower and higher sea salt concentrations. A similar scenario was exhibited by marine bacterium *P. carrageenovora* ASY5, which did not produce a substantial amount of ι-carrageenase at higher or lower sea salt concentration [31].

### 3.5. Optimal Nitrogen Source

**Figure 5:**
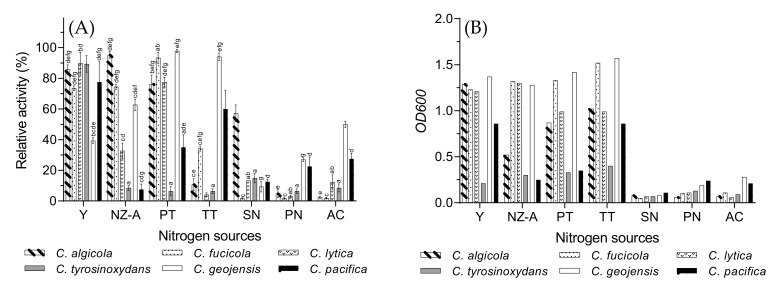
The effects of different nitrogen sources on *Cellulophaga* species on (**A**) ι-carrageenase production and (**B**) cell growth presented as optical density (*OD_600_*). Values of Figure 5A are expressed as mean ± SEM (n = 4). Data comparison was performed against nitrogen sources. *a* = when compared with yeast extract (Y), *b* = when compared with NZ-amine (NZ-A), *c* = when compared with peptone (PT), *d* = when compared with tryptone (TT), *e* = when compared with sodium nitrate (SN), *f* = when compared with potassium nitrate (PN), *g* = when compared with ammonium chloride (AC). Different lowercase letters on the bars indicate statistically significant differences from respective group using ANOVA, followed by Tukey’s comparison test (*p* < 0.05).

In their study, Xiao et al. stated the necessity of a nitrogen source in the fermentation medium for carrageenase production, as it provides substances required for protein synthesis, nucleic acid, and nitrogen metabolites [31]. In this study, different organic (yeast extract, NZ-amine, peptone, tryptone) and inorganic (sodium nitrate, potassium nitrate, ammonium chloride) nitrogen sources were used to stimulate the production of ι-carrageenase by the *Cellulophaga* species. The organic nitrogen sources were more effective than the inorganic ones in terms of ι-carrageenase synthesis (Figure 5A). The overall bacterial growth also appeared to be much higher in the presence of organic nitrogen sources (Figure 5B). Zhou et al. also reported higher κ-carrageenase production from a *Pseudoalteromonas*-like bacterium, WZUC10, with the presence of organic nitrogen sources in the fermentation medium [38]. Besides, Muzyed et al. and Xiao et al. reported yeast extract as the optimal nitrogen source for ι-carrageenase production by *C. baltica* and *P. carrageenovora* ASY5, respectively [15,31]. Among the six tested species, *C. geojensis* and *C. pacifica* displayed noticeable growth and enzyme synthesis in the presence of potassium nitrate and ammonium chloride. Surprisingly, very little growth and enzyme production was observed in the presence of sodium nitrate, which could indicate that potassium ions also play an important role in terms of bacterial growth and ι-carrageenase synthesis by *C. geojensis* and *C. pacifica*. Since both species belong to the same aquatic environment in the Sea of Japan, where the average salinity is 34 PSU [39], their metabolic adaptation with the same environment and water salinity could potentially explain their similar behavior.

However, a higher number of *Cellulophaga* strains showed maximum carrageenase activity in the presence of yeast extract. Although *C. fucicola* and *C. geojensis* had the highest value of peptone, they also produced a satisfactory amount of carrageenase in the presence of yeast extract. Therefore, yeast extract was used as the optimal nitrogen source for the next experiments.

### 3.6. Optimal Yeast Extract Concentration

**Figure 6:**
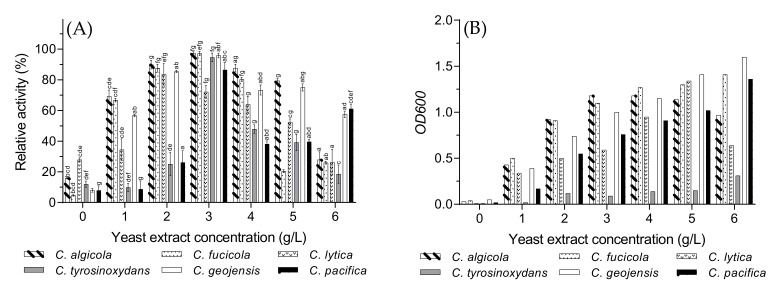
The effects of different yeast extract concentrations on the *Cellulophaga* species on (**A**) ι-carrageenase production and (**B**) cell growth presented as optical density (*OD_600_*). Values of Figure 6A are expressed as mean ± SEM (n = 4). Data comparison was performed against yeast concentrations. *a* = when compared with 0 g/L, *b* = when compared with 1 g/L, *c* = when compared with 2 g/L, *d* = when compared with 3 g/L, *e* = when compared with 4 g/L, *f* = when compared with 5 g/L, *g* = when compared with 6 g/L. Different lowercase letters on the bars indicate statistically significant differences from respective group using ANOVA, followed by Tukey’s comparison test (*p* < 0.05).

Yeast extract, at different concentrations, was selected as an independent research objective based on the maximum amount of carrageenase production by the tested bacterial strains. As shown in Figure 6B, the presence of yeast extract in the fermentation medium was essential for the growth of the *Cellulophaga* species, and different concentrations were tested in the range of 0–6 g/L. Although the growth rate kept rising for most of the tested species with a higher concentration (Figure 6B), it showed an adverse effect on carrageenase production. Despite the upward trend of enzyme production with higher yeast extract concentration, enzyme production was reduced marginally after reaching its optimal value at 3 g/L (Figure 6A). This result suggests the pendency of carrageenase production on yeast extract concentration. Some previous studies have revealed similar results. In one study, the production of ι-carrageenase from *P. carrageenovora* was limited by higher and lower concentrations of yeast extract [31], whereas researchers have shown that the *C. baltica* strain produced maximum amount of ι-carrageenase by 4 g/L of yeast extract in the fermentation medium [15]. The production of κ-carrageenase also decreased significantly with higher yeast extract concentration by *Thalassospira* sp. Fjfst-332 where the optimal concentration was 1 g/L [18]. The notable drop in enzyme production after exceeding a certain concentration of yeast extract could be caused by the excessive amount of easily accessible nutrients which inhibit carrageenase production. Thus, the lack of easily fermentable nutrients may stimulate the production of enzymes capable of degrading the biopolymers that are not the primary carbon source for a specific microorganism.

### 3.7. Optimal Furcellaran Concentration

The presence of some specific carbon sources in the fermentation medium act as inducers for the carrageenase production [40,41]. Different types of carrageenans (κ-, ι-, and λ-carrageenans) and furcellaran have been previously used to stimulate the carrageenase production [31,38]. Xiao et al. reported higher ι-carrageenase synthesis in the presence of ι-carrageenan from *P. carrageenovora* ASY5 [31]. Muzyed et al. also found a satisfactory amount of ι-carrageenase production from *C. baltica* in the presence of commonly used polysaccharides such as κ- and ι-carrageenans [15However, their highest enzyme production rate was recorded in the presence of furcellaran (a hybrid κ/β-carrageenan) in the fermentation medium from *C. baltica* [15]. Since all the studied species belong to the same genus (*Cellulophaga*), furcellaran was used as a carbon source in addition to yeast extract in this study for the stimulation of the enzyme production. It could be hypothesized that the hybrid nature of the galactan plays an important role in initiating of the production of various carrageenolytic enzymes, as such polysaccharides are often more difficult to degrade, and simultaneous action of different enzymes is required to cleave them down to the oligomeric size.

**Figure 7:**
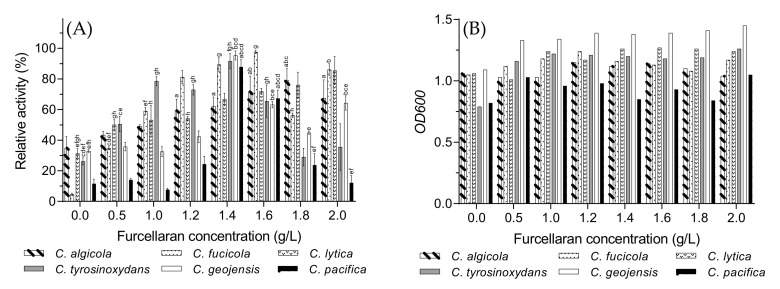
The effects of different furcellaran concentrations on the *Cellulophaga* species on (**A**) ι-carrageenase production and (**B**) cell growth of the *Cellulophaga* species presented as optical density (*OD_600_*). Values of Figure 7A are expressed as mean ± SEM (n = 4). Data comparison was performed against furcellaran concentrations. *a* = when compared with 0 g/L, *b* = when compared with 0.5 g/L, *c* = when compared with 1 g/L, *d* = when compared with 1.2 g/L, *e* = when compared with 1.8 g/L, *f* = when compared with 2.0 g/L. Different lowercase letters on the bars indicate statistically significant differences from respective group using ANOVA, followed by Tukey’s comparison test (*p* < 0.05).

The ι-carrageenase production rate gradually increased with furcellaran concentration, and the maximum value was obtained at 1.4–1.6 g/L for most of the bacteria (Figure 7A). Figure 7B reveals that bacterial growth was not significantly affected by the addition of furcellaran. Due to high viscosity, which may potentially impair the growth of the bacteria [15], the furcellaran concentration was limited to 2 g/L in this study.

Although *C. lytica* and *C. fucicola* are situated quite far from each other in the phylogenetic tree (Figure 1), their enzyme production rate interestingly remained rather stable even after their optimal value. However, the production of ι-carrageenase significantly decreased for *C. tyrosinoxydans*, *C. geojensis*, and *C. pacifica* after 1.4 g/L of furcellaran in the fermentation medium. Hence, the optimal concentration of furcellaran was selected to be 1.4 g/L.

### 3.8. Effect of Temperature and pH on Enzymatic Degradation

**Figure 8:**
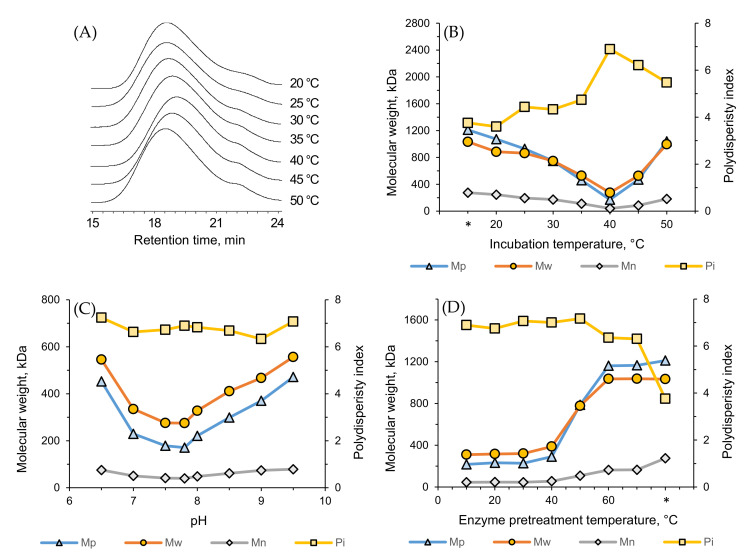
The effects of temperature and pH on ι-carrageenan degradation by the ι-carrageenase extracted from *C. algicola.* (**A**) Size-exclusion chromatography profiles and (**B**) molecular weights and polydispersity indexes after enzymolysis at different temperatures, (**C**) molecular weights and polydispersity indexes of substrates depending on the pH of the enzymolysis media, and (**D**) after enzymolysis with preheated enzymes. (*) in Figure 8B,D indicates the untreated ι-carrageenan sample.

The degradation of ι-carrageenan was performed by ultrafiltered enzymes (described in Section 2.2.5) extracted from the *Cellulophaga* species. As expected, *C. algicola* showed the highest degradation ability (data not shown), since *C. algicola* also had the highest absolute activity among the *Cellulophaga* species (Figure 2). A comparative pattern was achieved using a wide range of incubation temperatures (20–50 °C). The peak average molecular weights (Mp) of the degraded ι-carrageenan varied between 171 kDa and 1073 kDa, while the weight average molecular weights (Mw) varied between 275 kDa and 997 kDa (Figure 8B). This difference was also visible in the size-exclusion chromatograms (Figure 8A). The Mp and Mw values for the untreated ι-carrageenan sample were 1211 kDa and 1033 kDa, respectively, with a polydispersity index (Pi) value of 3.76 (Figure 8B). The enzyme was most effective at 40 °C, resulting in the lowest Mp and Mw values of ι-carrageenan when the Pi was at the highest point (Figure 8B). Similar results have also been found in the previous literature. Jouanneau et al. efficiently conducted the degradation of ι-carrageenan using a similar enzyme from *Alteromonas fortis* at 40 °C [42], while Xiao et al. reported the optimal temperature of their ι-carrageenase from *Pseudoalteromonas carrageenovora* ASY5 at 40 °C [31].

The degradation of ι-carrageenan was measured at different pH values, and the enzyme was at its most active form at pH 7.5–8.0. Several studies have also revealed a similar range as the optimal pH for ι-carrageenase [43,44,45]. Figure 8C exhibits that the lowest Mp (171 kDa) and Mw (275 kDa) were obtained at pH 7.8, indicating the optimal pH value in terms of enzymatic degradation.

The enzyme was preheated at different temperatures ranging from 10–70 °C to determine its thermostability. Figure 8D reveals that the enzyme remained the most active at 10–40 °C, resulting in degraded ι-carrageenans with Mp values in the range of 217–290 kDa and Mw values in the range of 310–388 kDa. The enzyme activity started to drop rapidly after 40 °C, indicating the edge of stability, and was almost completely inactive after 60 °C. Such stability is slightly higher than for some previously characterized ι-carrageenases, such as the ι-carrageenases stability of *C. baltica* and *P. carrageenovora* ASY5, respectively. as Muzyed et al. and Xiao et al. stated that the thermostability range of their ι-carrageenase was 35 °C [15,31].

## 4. Conclusions

In this paper, the carrageenase secretion ability of six *Cellulophaga* species was studied, and all species produced a satisfactory amount of ι-carrageenase. Most of the bacteria needed 48 h of incubation time at 25 °C to produce the maximum amount of ι-carrageenase. *C. algicola* was the most potent carrageenase producer, followed by *C. fucicola* and *C. geojensis*. The presence of sea salt and furcellaran in the fermentation medium enhanced the enzyme production of the *Cellulophaga* species, with optimal values of 30 g/L and 1.4 g/L, respectively. Similar enzyme production pattern was observed for *C. algicola* and *C. fucicola* in different sea salt concentrations, and for *C. lytica* and *C. fucicola* in different furcellaran concentrations. Yeast extract was confirmed to be the optimal nitrogen source, and most of the species of bacterial yielded the highest amount of ι-carrageenase at 3 g/L of yeast extract. By enzymatic degradation, ultrafiltered ι-carrageenase could produce ι-carrageenan with the lowest molecular weight at 40 °C. The preheated enzyme remained active at 10–40 °C, and the optimal pH was determined as 7.8. The determination and study of the carrageenase activity with optimal fermentation conditions by these *Cellulophaga* species could contribute to the industrial production of oligosaccharides. Therefore, the discovered enzymes need further detailed investigation to utilize their industrial potential more efficiently.

## Data Availability

Not applicable.
